# An Interdisciplinary Review of the Zygomaticus Muscles: Anatomical Variability, Imaging Modalities, and Clinical Implications

**DOI:** 10.3390/jcm14124110

**Published:** 2025-06-10

**Authors:** Ingrid C. Landfald, Łukasz Olewnik

**Affiliations:** Department of Clinical Anatomy, Mazovian Academy in Plock, 2, 09-402 Plock, Poland; ingridceciliee@gmail.com

**Keywords:** zygomaticus major, zygomaticus minor, facial muscles, anatomical variation, smile biomechanics, mimetic muscles, facial expression, MRI, ultrasonography, 3D imaging, AI segmentation, Duchenne smile, facial reconstruction

## Abstract

The zygomatic major and zygomatic minor muscles play a central role in facial expression, particularly in generating the smile, one of the most essential forms of human nonverbal communication. While their function is widely recognized, the anatomical variability in these muscles remains underexplored in both clinical and surgical settings. This review provides a comprehensive, interdisciplinary analysis of the zygomaticus musculature, integrating classical anatomical insights with recent advances in imaging, developmental biology, and artificial intelligence-based analysis. By examining data from cadaveric dissection, MRI, ultrasonography, and 3D photogrammetry, we identify key morphological differences with potential clinical relevance. A novel five-type morphological classification is proposed, based on differences in the number of muscle bellies (i.e., belly number), accessory structures, insertion patterns, and population-based variation. This classification aims to offer a more functionally relevant and clinically applicable framework for use in facial surgery, aesthetic procedures, and forensic reconstruction. By moving beyond the simplistic binary categorizations that have historically defined zygomaticus morphology, this review highlights the need for a personalized approach to facial anatomy, tailored to individual morphological variation. The proposed framework may assist in refining surgical planning, improving outcomes in facial reanimation, and enhancing diagnostic accuracy in both radiological assessment and preoperative planning. By moving beyond traditional binary categorizations, this review highlights the need for a personalized approach to facial anatomy, tailored to individual morphological variations. The proposed framework may assist in refining surgical planning, improving outcomes in facial reanimation, and advancing diagnostic precision in facial imaging. A total of 75 peer-reviewed articles were selected based on a targeted search in PubMed, Scopus, and Web of Science (1995–2024).

## 1. Introduction

Facial muscles play a crucial role in emotional expression, social interaction, and nonverbal communication by enabling a wide range of nuanced facial movements [1,2]. Among these, the mimetic muscles innervated by the facial nerve (cranial nerve VII) attach directly to the skin, facilitating the dynamic modulation of facial contours [3].

The scientific study of facial expressions, initiated by Darwin [4], has emphasized their evolutionary role in emotional signaling. Subsequent research confirmed the universality of core emotions (e.g., happiness, sadness, anger), while highlighting cross-cultural differences in expression intensity and interpretation [5,6,7,8,9].

Mimicry, or the subconscious replication of facial expressions, fosters empathy and emotional synchronization [10,11]. The ZMa is particularly critical in producing genuine (Duchenne) smiles—markers of sincerity and social bonding [1,5,12,13,14,15]. Alongside the ZMi, these muscles elevate the mouth corners and contribute to facial symmetry and expression aesthetics [12,16,17,18,19,20].

Anatomical variability in the ZMa and ZMi, including differences in origin, insertion, and muscle belly configuration, has gained increasing attention in clinical and research contexts [19,20,21,22,23]. Such variability can significantly influence outcomes in facial surgery, diagnostics, and aesthetic procedures. Modern imaging tools like MRI, ultrasonography (US), and CT aid in detecting these variations, though interpretation can remain challenging [24,25].

Anthropologists also rely on detailed anatomical knowledge of facial musculature for population studies, forensic reconstructions, and evolutionary analysis [26,27,28].

This review integrates anatomical, radiological, clinical, and developmental data to provide an updated, interdisciplinary synthesis of zygomaticus muscle morphology. In doing so, it introduces a novel five-type morphological classification, addressing the gap in the current literature that lacks a unified, clinically relevant model for describing these muscles. By combining traditional anatomical dissection with modern imaging and AI segmentation data, this review aims to answer how structural variability in ZMa and ZMi can affect facial function, aesthetics, and surgical outcomes. It seeks to establish a foundation for personalized anatomical approaches in facial surgery, diagnostics, and anthropological reconstruction [19,20,21].

## 2. Classical Anatomy of the Zygomaticus Muscles

### 2.1. Zygomatic Major Muscle: Anatomical Course, Attachments, and Function

The ZMa muscle is one of the primary muscles of facial expression, playing a pivotal role in controlling the movements related to smiling and expressing positive emotions [16]. Classically described as a slender, elongated muscle, ZMa originates proximally from the lateral surface of the zygomatic bone, specifically from the region anterior to the zygomaticotemporal suture and superior to the origin of the ZMi [15], as seen in Table 1.

Its fibers typically extend inferomedially toward the oral commissure, inserting distally into the dermal and muscular tissues at the angle of the mouth and blending extensively with fibers of the orbicularis oris muscle and superficial muscular aponeurotic system (SMAS) [29].

ZMa often exhibits variability in its anatomical course, length, and points of insertion, with some fibers occasionally intermingling with adjacent muscles such as the levator anguli oris or the risorius [23]. Despite this variability, its primary functional role remains consistent, involving the elevation and lateral retraction of the angle of the mouth. This characteristic action contributes significantly to facial expressions associated with smiling, laughter, and emotional authenticity, differentiating genuine (Duchenne) smiles from non-Duchenne smiles [5,7,12].

The functional and anatomical characteristics of ZMa have essential implications in clinical practices, including facial reconstructive surgery, aesthetic procedures, and diagnostic imaging. The accurate identification of ZMa’s anatomical landmarks and potential variations is crucial for minimizing risks during interventions such as botulinum toxin injections, facelifts, and reconstructive surgeries aimed at correcting facial asymmetry and paralysis [21,30,31]. Therefore, precise anatomical knowledge of the ZMa muscle is indispensable in both clinical and research contexts, ensuring effective diagnosis, treatment planning, and optimal procedural outcomes.

### 2.2. Zygomatic Minor Muscle: Anatomical Description and Functional Differences

The ZMi muscle, although anatomically smaller and more variable compared to ZMa, contributes distinctly to the nuanced control of facial expressions, particularly in movements involving the elevation and subtle retraction of the upper lip [16]. Classically, the ZMi originates from the anterior aspect of the zygomatic bone, slightly medial and superior to the origin of ZMa. Its fibers course inferomedially, inserting into the muscular and dermal layers of the upper lip, frequently blending with the levator labii superioris muscle and sometimes directly intermingling with fibers of the orbicularis oris muscle [15].

The anatomical presence and exact insertion points of ZMi demonstrate significant variability, with frequent occurrences of absent, duplicated, or accessory muscle bands, which are commonly reported in anatomical dissections and radiological assessments [23,29]. Such variations can influence individual differences in facial expressions, smile aesthetics, and overall facial symmetry [21].

Functionally, while ZMa primarily facilitates lateral and superior displacement of the oral commissure (characteristic of smiling and laughter), ZMi predominantly elevates the upper lip, playing a supportive role in generating subtle emotional expressions and aiding more precise movements during facial gestures [16]. This functional distinction, although subtle, is essential in differentiating ZMi from ZMa during clinical assessments and surgical planning. For instance, selective paralysis or targeted treatments affecting ZMi may specifically alter the upper lip contour and subtle aspects of emotional expression without significantly affecting broader smile mechanics [30,31].

The clinically detailed anatomical understanding and identification of ZMi’s anatomical variability are crucial during aesthetic procedures, reconstructive facial surgeries, and diagnostic imaging interpretation to ensure precise treatment planning and optimized patient outcomes [21].

### 2.3. Anatomical Relationships of Zygomatic Major Muscle and Zygomatic Minor Muscle with Adjacent Midfacial Mimetic Structures (Levator Labii Superioris, Orbicularis Oculi, SMAS)

The anatomical and functional interactions of ZMa and ZMi with adjacent mimetic structures in the midface significantly influence facial expressions and are vital to surgical and clinical considerations. These relationships predominantly include connections with the levator labii superioris muscle, orbicularis oculi muscle, and the superficial musculoaponeu (SMAS), forming a complex and dynamic anatomical network responsible for coordinated facial movements [16].

The ZMi muscle closely interacts with the levator labii superioris, often merging at its distal attachments within the upper lip region. This anatomical relationship contributes notably to the combined elevation and subtle retraction of the upper lip, playing a pivotal role in nuanced emotional expressions [29]. Such close muscular integration necessitates careful identification and preservation during aesthetic procedures or facial reconstructions to maintain natural expression and lip mobility [21].

ZMa, by comparison, shows strong anatomical continuity with the superficial musculoaponeurotic system (SMAS), particularly at the level of its insertion around the oral commissure. This relationship enhances the transmission of muscular forces, contributing significantly to lateral facial tension, the elevation of the oral commissure, and facial skin movement during expressions like smiling and laughing [11,23]. The integrity of this relationship is crucial during facelift surgeries and other cosmetic procedures involving the manipulation or repositioning of SMAS, directly influencing postoperative facial dynamics and aesthetic outcomes [11,21].

Additionally, the anatomical relationship between ZMa, ZMi, and the orbicularis oculi muscle is functionally important in the coordination of genuine facial expressions, such as the Duchenne smile. The concomitant activation of ZMa with the orbicularis oculi muscle is well documented, reflecting authentic emotional expression that involves the simultaneous elevation of the mouth corners and the formation of characteristic “crow’s feet” around the eyes [7,12]. Anatomical continuity between these muscles via fascial connections facilitates synchronous movement and supports complex expressions, underscoring their collective significance in mimetic function [15,16].

A comprehensive understanding of these anatomical relationships and their implications is essential in clinical contexts, ranging from reconstructive facial surgery and aesthetic procedures to advanced imaging assessments, enabling clinicians to preserve or restore optimal facial expressiveness and symmetry [30,31].

### 2.4. Blood Supply and Innervation: Topographic Relevance for Surgical and Injectable Procedures

The ZMa and ZMi muscles receive their blood supply predominantly from branches of the facial artery and its extensive anastomotic network with the infraorbital artery, transverse facial artery, and buccal artery [16,32,33]. Such a rich vascular network ensures the adequate perfusion necessary for their dynamic functional activity. Venous drainage parallels arterial supply, primarily via tributaries of the facial vein, thus forming an essential anatomical consideration during facial surgery and minimally invasive procedures [21,33].

The innervation of ZMa and ZMi is provided exclusively by branches of the facial nerve (cranial nerve VII), specifically via the zygomatic and buccal branches. These branches traverse through the SMAS, emerging from the parotid gland and coursing anteriorly across the midface region [15,16]. Precise anatomical knowledge of these neural pathways is crucial for preserving muscular function during surgical procedures such as facelift surgeries, facial reanimation, or trauma reconstruction, as inadvertent nerve injury can result in significant morbidity, manifesting as paralysis, facial asymmetry, or impaired emotional expression [16,23,30].

Understanding the detailed anatomy of these vascular and neural structures is equally critical for procedures involving injectable substances, such as botulinum toxin or dermal fillers. Accurate identification of nerve trajectories and vascular territories helps to avoid complications, including hematomas, inadvertent arterial injection, ischemia, or neuromuscular dysfunction [21,31,34]. Detailed mapping through advanced imaging modalities, such as high-resolution US and MRI, is becoming increasingly recommended to precisely delineate these anatomical landmarks and facilitate safer clinical interventions [19,35].

Thus, comprehensive topographical knowledge regarding the vascularization and innervation of ZMa and ZMi is imperative for clinicians performing surgical or injectable facial procedures, significantly enhancing procedural safety, reducing risk, and improving therapeutic and aesthetic outcomes [21].

## 3. Embryology and Genetic Basis

### 3.1. Development of Mimetic Muscles from the Second Pharyngeal Arch

Facial mimetic muscles, including ZMa and ZMi, embryologically originate from the mesoderm of the second pharyngeal (branchial) arch, also known as the hyoid arch, during early embryonic development [16]. The second arch mesenchyme, composed primarily of neural crest-derived mesenchymal cells along with paraxial mesodermal cells, migrates into specific regions of the future face, giving rise to distinct groups of facial muscles through complex signaling interactions [2,36].

During the fourth to eighth weeks of embryogenesis, the proliferation and differentiation of these mesenchymal cells lead to the formation of the facial musculature under the influence of coordinated gene expression patterns and signaling pathways, such as fibroblast growth factors (FGFs), sonic hedgehog (Shh), and Wnt signaling [2,10,37,38]. By approximately the seventh week of gestation, the mesenchyme of the second pharyngeal arch specifically differentiates into the primordial facial muscles, including ZMa and ZMi, establishing their anatomical positions and basic morphological features [2].

The differentiation of facial muscles is intricately linked with the migration and branching pattern of the facial nerve (cranial nerve VII), which innervates all muscles derived from the second pharyngeal arch mesenchyme. This relationship underscores the critical importance of coordinated embryonic development, as any disruptions in mesenchymal differentiation, neuronal migration, or signaling processes may lead to congenital malformations or functional anomalies in the mimetic muscles, potentially resulting in clinical syndromes such as Treacher Collins, Möbius, or Goldenhar syndromes [2,27].

Comprehensive knowledge of the embryological development and genetic regulation underlying the formation of ZMa, ZMi, and related facial musculature provides essential insights into both normal facial morphogenesis and pathological conditions, informing clinical approaches to congenital facial anomalies and reconstructive surgical interventions [27,36].

### 3.2. Sequence of Facial Muscle Differentiation and Integration with the Facial Nerve (CN VII)

The differentiation of facial muscles, including ZMa and ZMi, is closely coordinated with the development of the facial nerve (cranial nerve VII), reflecting a tightly regulated embryological process. During embryogenesis, cranial nerve VII emerges from the rhombencephalon region of the developing hindbrain and extends its fibers toward the mesenchymal condensations of the second pharyngeal arch, ultimately innervating all muscles derived from this arch [16,39].

Initially, around the fourth week of embryonic development, facial nerve motor fibers begin to migrate into the second pharyngeal arch region, guiding subsequent muscle differentiation and alignment through a combination of chemoattractant and chemorepulsive signaling molecules [27,39]. By approximately the seventh to eighth week, mesenchymal cells within the second arch undergo differentiation into myoblasts, subsequently fusing to form multinucleated muscle fibers, a process largely regulated by gene expression patterns involving MyoD, Pax3, Pax7, and related transcription factors [36,38].

Concurrently, the precise timing and spatial distribution of facial nerve branching are crucial, as motor neurons establish synaptic connections with developing muscle fibers. This neuromuscular integration ensures the functional specialization of mimetic muscles, such as ZMa and ZMi, facilitating their precise voluntary and emotional movements after birth [2,27]. Disruptions in this integrated development, due either to genetic mutations or environmental factors, can result in significant facial anomalies, including congenital facial paralysis and various craniofacial syndromes, highlighting the critical importance of the tightly regulated developmental sequence [27].

Thus, detailed understanding of the coordinated developmental timeline and integration of ZMa, ZMi, and other facial muscles with cranial nerve VII is essential for elucidating both normal developmental processes and pathologies involving facial expression musculature, guiding effective diagnostic and therapeutic approaches in clinical embryology and facial reconstructive practices [39].

### 3.3. Key Genes in Facial Muscle Morphogenesis: Tbx1, MyoD, Pax3, and Pitx2

The morphogenesis of facial musculature, including ZMa and ZMi, is tightly regulated by an intricate network of genes, which ensure the precise spatial and temporal differentiation of facial muscles during embryonic development [36,38]. Among the pivotal regulatory genes, Tbx1, MyoD, Pax3, and Pitx2 are particularly significant, playing distinct but interrelated roles in the myogenic differentiation pathway that forms facial musculature from mesodermal progenitor cells derived primarily from the second pharyngeal arch [27,39].

Tbx1, a T-box transcription factor gene, plays critical roles in early craniofacial patterning, pharyngeal arch segmentation, and myogenic differentiation within the second pharyngeal arch. Mutations or deletions of Tbx1 result in severe craniofacial malformations and muscle anomalies, prominently observed in conditions such as DiGeorge syndrome and velocardiofacial syndrome, underlining its crucial regulatory role during the early stages of facial muscle development [40,41].

MyoD, a myogenic regulatory factor belonging to the basic helix–loop–helix (bHLH) family of transcription factors, acts downstream of initial arch patterning genes and is directly responsible for triggering muscle-specific gene expression. It initiates the differentiation of mesenchymal progenitor cells into myoblasts, subsequently promoting their fusion into multinucleated muscle fibers, crucial for the proper formation of facial muscles such as ZMa and ZMi [36,38].

Pax3, a paired box transcription factor gene, contributes significantly to the early specification, survival, and migration of myogenic progenitor cells. It also regulates the expression of MyoD, thereby indirectly influencing myogenesis and muscular differentiation in facial regions. Dysfunction of Pax3 has been linked to severe congenital craniofacial muscular anomalies, exemplified by conditions such as Waardenburg syndrome, emphasizing its indispensable role in facial muscle development [38,42].

Pitx2, a paired-like homeodomain transcription factor gene, further influences facial muscle patterning by regulating left–right asymmetry and the regional specification of facial muscle groups. Its expression is crucial in defining muscular differentiation boundaries, ensuring proper orientation and positional identity of developing facial muscles, including ZMa and ZMi. Mutations in Pitx2 are notably implicated in craniofacial disorders such as Axenfeld-Rieger syndrome, demonstrating the importance of precise gene regulation for normal facial morphogenesis [27].

Understanding the coordinated actions and regulatory interactions of these key genes offers critical insights into the normal and pathological development of facial musculature, providing essential knowledge for interpreting developmental anomalies and informing clinical management strategies for congenital facial muscle disorders [27,38,39].

Recent studies have linked the expression of key myogenic regulatory genes, such as *Tbx1* and *MyoD*, to specific craniofacial muscle patterning, suggesting that dysregulation during somitomere-derived mesoderm differentiation may contribute to observed morphological variants of the ZMa and ZMi [43,44].

### 3.4. Developmental Variability and Its Clinical Relevance in Disorders Such as Treacher Collins, Williams, and Down Syndromes

Developmental variability in facial muscle morphogenesis, including variations in ZMa and ZMi, significantly influences the clinical presentation and severity of congenital disorders affecting craniofacial structures. Syndromes such as Treacher Collins, Williams, and Down syndrome exemplify conditions in which disrupted genetic and embryological pathways markedly impact facial muscle development, leading to distinctive facial phenotypes and functional impairments [27,39].

Treacher Collins syndrome (TCS), an autosomal dominant craniofacial disorder primarily resulting from mutations in the TCOF1 gene, is characterized by hypoplasia of the zygomatic bones, mandibular deficiencies, and pronounced malformations of second pharyngeal arch-derived structures, including mimetic muscles such as ZMa and ZMi. The disruption in neural crest cell migration and proliferation that characterizes TCS profoundly affects muscular differentiation and facial nerve integration, frequently leading to muscular hypotrophy, facial asymmetry, and impaired facial expressions [45].

Williams syndrome, a developmental disorder resulting from deletions on chromosome 7q11.23 encompassing the elastin (ELN) gene, displays characteristic dysmorphic features and pronounced variability in facial musculature, impacting muscles including ZMa and ZMi. Patients often exhibit distinctive facial expressions described as “elfin facies,” marked by prominent zygomatic regions, broad smiles, and subtle muscular hyperactivity. Such variability in facial muscular morphology contributes significantly to the characteristic social engagement and expressive facial behaviors commonly observed in affected individuals [24,46].

Down syndrome (trisomy 21), another well-studied genetic disorder, is characterized by extensive developmental variability and distinctive craniofacial phenotypes. Individuals commonly exhibit facial muscular hypotonia, reduced muscle tone, and altered muscular morphology, affecting the ZMa, ZMi, and related facial musculature. These muscular alterations often lead to characteristic expressions and functional impairments, including difficulties in feeding and speech articulation, and a reduced ability to perform coordinated facial movements [6,47].

Understanding the embryological and genetic basis underlying these developmental variations in facial muscle formation is crucial in improving clinical management, diagnostic precision, and surgical interventions. It provides clinicians and surgeons with critical insights into patient-specific anatomical presentations, facilitating individualized therapeutic approaches aimed at optimizing functional outcomes and enhancing quality of life for affected individuals [27,39,46].

In addition to the syndromes already discussed, craniofacial myopathy and myotonic dystrophy have been associated with impaired facial muscle development, potentially altering the size, symmetry, or functional capacity of the zygomaticus muscles. These conditions underscore the need for individualized anatomical assessment in patients undergoing facial surgery or diagnostics [48].

A summary of the key genetic and syndromic conditions affecting zygomaticus muscle morphology is presented in Table 2.

## 4. Morphological Variations

### 4.1. Historical Reports

Historical accounts concerning morphological variations in ZMa and ZMi muscles provide valuable foundational knowledge and demonstrate a longstanding awareness of anatomical diversity. Early anatomical texts by Macalister, Calori, Testut, Le Double, and Gruber have extensively documented the complexity of and variability in facial musculature, highlighting their clinical relevance.

Macalister [49] provided one of the earliest comprehensive descriptions of muscular variations, including extensive observations of ZMa and ZMi. His detailed documentation illustrated cases of accessory muscle slips, duplication, and bifurcation of these muscles, as well as atypical insertion points that influenced facial expression dynamics [17,23,49].

Similarly, Joseph Le Double [50] presented an exhaustive exploration of human muscular variations, noting significant variability in ZMa and ZMi morphology. His meticulous dissections frequently demonstrated duplicated and accessory zygomaticus muscles, emphasizing their prevalence and potential clinical impact [50].

Testut [26], in his influential anatomical treatise, also described numerous variations related to ZMa and ZMi. He reported cases of absent or rudimentary ZMi muscles and supplementary muscular slips that considerably influenced facial expression and symmetry. These observations underscored the inherent anatomical complexity present within facial musculature [26].

Calori [51] and Gruber [52], prominent anatomists known for their extensive contributions to the understanding of muscular variability, provided significant early observations of anomalous muscular formations, laying the groundwork for subsequent studies. Although specific accounts regarding ZMa and ZMi from their original texts are less frequently cited, their general work significantly informed the anatomical literature and contributed to the broader awareness of muscular diversity [17,23,51,52].

Collectively, these historical reports have been instrumental in establishing a foundational understanding of facial muscular anatomy, underscoring variability that remains clinically significant today in facial reconstructive surgery, diagnostic radiology, and anthropological research [1,22,29,53,54,55].

### 4.2. Description of Main Morphological Variants

#### 4.2.1. Variation in Muscle Length and Width

Significant variability in the length and width of ZMa and ZMi muscles has been frequently documented in anatomical and radiological studies, reflecting the inherent diversity in facial muscular morphology [1,22,29,53,55]. Such variations have direct implications for facial expressivity, muscular function, and surgical outcomes.

Spiegel and DeRosa [55] provided detailed measurements regarding the precise location of ZMa in relation to orbicularis oculi (OOc) and levator labii superioris muscles, emphasizing individual variability in the vertical and lateral positioning of these muscles. Elvan et al. [1] further described variability in muscle shape, reporting ribbon-like (51%), fan-shaped (34%), and bifid (13%) types, highlighting morphological diversity relevant for surgical planning.

Multiple morphological variants of the zygomaticus muscles have been identified, ranging from bifid to multibellied formations, accessory muscle bands, and unusual insertion patterns. These variants exhibit distinct anatomical features and varying prevalence across studies, often carrying important clinical implications. Table 3 provides an overview of the main morphological types, their characteristics, and reported frequencies based on the current literature.

#### 4.2.2. Variation in Number of Muscle Bellies (Single, Double, and Multiple Bellies)

Considerable morphological variability in the ZMa and ZMi muscles has been reported in relation to the number of muscle bellies, reflecting significant anatomical diversity. Typically, the ZMa and ZMi muscles present as single-bellied structures, yet anatomical dissections frequently reveal variations, including double-bellied (bifid) or even multibellied formations [1,16,22,29,53].

Pessa et al. [29] specifically investigated the bifid variant of the ZMa, reporting an incidence of 34% among their sample. In these cases, ZMa typically began as a single structure from the zygomatic bone and subsequently bifurcated into superior and inferior bellies. The inferior belly occasionally demonstrated a dermal insertion, correlating with the presence of facial dimples during smiling. Phan and Onggo [54] further examined the prevalence of the bifid ZMa through a meta-analysis, reporting an overall occurrence of 22.7%, with significant ethnic differences.

Additionally, Elvan et al. [1] reported the bifid form of ZMa in 13% of their cases, reinforcing previous data and demonstrating significant clinical relevance regarding facial expressions and surgical implications.

#### 4.2.3. Additional Muscle Bands (e.g., ZMi Duplicatus, Accessory Zygomaticus)

Additional muscle bands associated with ZMa and ZMi, such as ZMi duplicatus and accessory zygomaticus muscle slips, represent notable morphological variations frequently documented in anatomical studies and cadaveric dissections [1,22,55]. Interdigitations between ZMa and OOc described by Spiegel and DeRosa [55] and varied spatial relationships highlighted by Elvan et al. [1] further emphasize the complexity of muscular interactions in the midfacial region.

#### 4.2.4. Atypical Insertion Points (e.g., Upper Lip, Orbicularis Oris, Cheek)

Atypical insertion points of ZMa and ZMi muscles constitute significant anatomical variations frequently identified during cadaveric dissections and imaging studies. Variations in muscle insertions substantially influence facial expression biomechanics, symmetry, and the outcomes of facial surgical procedures [1,22,29,53,55].

Elvan et al. [1] specifically reported that ZMa frequently overlapped or neighbored the parotid duct, with potential implications for surgical interventions. Spiegel and DeRosa [55] similarly emphasized variability in insertion points concerning the relationship between ZMa and adjacent facial muscles, underlining the clinical importance of accurate anatomical knowledge to ensure optimal surgical outcomes.

Comprehensive awareness of these morphological variations aids clinicians and surgeons in optimizing procedures, improving facial symmetry and expression, and minimizing postoperative complications.

### 4.3. Prevalence of Morphological Variations—Data from Cadaveric Studies, MRI, and Ultrasonography

The prevalence of morphological variations in ZMa and ZMi muscles has been extensively documented across various modalities, including cadaveric dissections, MRI, and US, reflecting substantial anatomical diversity with significant clinical implications [1,22,29,53,54,55].

Cadaveric studies consistently highlight considerable variation in the presence of bifid ZMa, ranging from 13% to 34% [1,29,54]. Specifically, Pessa et al. [29] reported a 34% prevalence of bifid ZMa in a sample of 50 cadaveric hemifaces, with distinct superior and inferior bellies, the latter occasionally inserting directly into the skin, correlating with facial dimples. Phan and Onggo [54], conducting a meta-analysis of multiple cadaveric studies, found an overall prevalence of bifid ZMa at approximately 22.7%, highlighting notable ethnic variability.

Further research by Elvan et al. [1] identified not only bifid variants (13%) but also other shape variations such as ribbon-like (51%) and fan-shaped forms (34%). Additionally, the anatomical relationship between ZMa and adjacent structures, such as the orbicularis oculi muscle (OOc) and parotid duct, exhibited significant variability, emphasizing the necessity for careful consideration during facial surgeries to minimize complications [1,55].

Imaging studies, particularly MRI and US, have corroborated findings from cadaveric dissections, providing additional clarity on the in vivo prevalence and morphological characteristics of facial muscles. MRI analyses have facilitated the detailed mapping of muscular anatomy, clearly demonstrating variability in muscle length, width, and insertion points, thereby influencing surgical and aesthetic outcomes [21]. The US, due to its real-time dynamic imaging capability, offers valuable insights into functional anatomy, allowing clinicians to visualize variations and asymmetries in ZMa and ZMi muscles during facial expression, further validating anatomical findings from cadaveric research [56].

Collectively, data from cadaveric dissections, MRI, and US underscore the widespread presence and clinical relevance of ZMa and ZMi muscle variations. Accurate knowledge of these anatomical details is crucial for clinicians and surgeons involved in facial reconstructive and aesthetic procedures, guiding personalized approaches to enhance functional and aesthetic outcomes and reduce procedural complications [1,22,29,53,54,55].

### 4.4. Population Variability—Differences Related to Sex, Age, and Ethnicity

Population variability in morphological characteristics of ZMa and ZMi muscles has been observed across numerous anatomical, radiological, and anthropological studies, emphasizing the influence of factors such as sex, age, and ethnicity on facial muscle anatomy [1,22,29,53,54,55].

Sex-based differences in facial musculature have been reported, though data specifically focusing on ZMa and ZMi remain relatively limited. However, general anatomical studies indicate that males typically possess more robust facial musculature with larger and thicker muscle fibers compared to females, likely due to hormonal and developmental differences influencing muscle mass and strength [16,21]. These differences have clinical significance in aesthetic procedures, such as filler injections and surgical planning, where tailored approaches are necessary to achieve optimal and natural outcomes [21].

Age-related variations in the morphology of ZMa and ZMi are primarily characterized by changes in muscle volume, elasticity, and functional efficiency. Progressive muscular atrophy and loss of facial muscle elasticity with aging have been documented extensively, influencing facial expressivity, symmetry, and the development of dynamic and static facial wrinkles [16,55]. Imaging and clinical studies underscore the need for age-specific considerations in facial rejuvenation and reconstructive procedures to ensure desirable aesthetic results and minimize complications [21,22,53].

Ethnic differences represent another critical dimension of population variability in ZMa and ZMi morphology. Phan and Onggo [54], in a meta-analysis, highlighted significant ethnic variability in the prevalence of bifid ZMa muscles, reporting the highest frequency in American populations (34%), followed by Asian (27.4%) and European (12.3%) groups. These findings emphasize the importance of considering ethnic variations during surgical planning and interventions, as anatomical diversity may influence the outcomes of facial surgery, the incidence of specific muscle anomalies such as bifid or accessory muscular bands, and aesthetic preferences [1,29,54,55].

Elvan et al. [1] further contributed valuable insights into ethnic variability by demonstrating distinct anatomical relationships between ZMa and adjacent structures such as the orbicularis oculi muscle (OOc) and parotid duct, variations potentially influenced by ethnicity and geographical origin. These variations necessitate individualized surgical approaches and careful clinical assessments to optimize procedural outcomes [1,22].

Overall, recognizing population-based variability in ZMa and ZMi anatomy specifically in relation to sex, age, and ethnicity is essential for clinical practice, facilitating personalized medical and surgical approaches, reducing procedural risks, and achieving improved cosmetic and functional outcomes.

### 4.5. Proposed Classifications of Variants in the Literature

Several classification systems have been proposed to categorize morphological variants of ZMa and ZMi. Pessa et al. [29] proposed a straightforward classification based on the presence of single or bifid ZMa bellies. Phan and Onggo [54] expanded this by highlighting ethnic-specific prevalence rates. Elvan et al. [1] introduced classifications based on ZMa shapes such as ribbon-like, fan-shaped, and bifid variants. Additionally, Spiegel and DeRosa [55] and Shim et al. [9] suggested classifications based on muscular interdigitations and relationships with neighboring muscles, offering clinically valuable landmarks and anatomical insights crucial for surgical interventions.

Collectively, these classification systems emphasize the importance of comprehensive anatomical knowledge to optimize clinical outcomes, enhance surgical precision, and minimize procedural complications [1,9,29,54,55].

### 4.6. Proposal of a New Morphological Classification

Based on the extensive anatomical, clinical, and radiological data reviewed above, a comprehensive and clinically relevant classification of ZMa and ZMi morphological variants is proposed. Previous studies have highlighted multiple aspects of variability, including muscle shape, the number of muscle bellies, accessory muscular bands, insertion points [1], relationships with neighboring anatomical structures, and population-based differences [9,29,54,55].

The proposed classification includes four principal dimensions:

1. Variations in muscle morphology (ZMa and ZMi):
  - Number of muscle bellies: single, bifid (double-bellied), multiple-bellied [1,29,54].
  - Muscle shape: ribbon-like, fan-shaped, fusiform [1].
2. Variations in muscle insertion:
  - Typical insertion: modiolus, oral commissure [16].
  - Atypical insertion: skin of the cheek (associated with facial dimples), orbicularis oris muscle, upper lip, or direct dermal attachment [55,57,58].
3. Anatomical relationship variants with neighboring structures:
  - Orbicularis oculi muscle (OOc): presence of muscular interdigitation or overlapping fibers [1,9,55].
  - Levator labii superioris muscle: direct muscle interconnections or adjacent courses [55].
  - Parotid duct: overlapped, adjacent, or separated from ZMa [1].
4. Population-based variability:
  - Ethnic differences: increased prevalence of bifid ZMa in Asian and American populations [9,54,58].
  - Sex-related differences: robustness and muscle volume variations between males and females [16,21].
  - Age-related changes: muscular atrophy, loss of elasticity, and changes in muscle volume affecting facial expression [16,21].

This proposed classification aims to provide a structured and practical framework for clinicians and anatomists. It underscores the importance of individualized anatomical assessment to optimize clinical outcomes in aesthetic, reconstructive, and anthropological applications, reducing surgical risks, and enhancing the precision of facial procedures [1,9,29,54,55,58]. Previous attempts to classify the morphology of the zygomaticus muscles have largely been limited in scope. Pessa et al. [29] introduced a binary classification based on the presence or absence of bifid ZMa morphology, noting its association with facial dimples and asymmetry. Phan and Onggo [54] extended this concept through a meta-analysis, revealing population-dependent prevalence but maintaining a focus solely on the bifid variant. Elvan et al. [1] proposed a shape-based system—categorizing ZMa into ribbon-like, fan-shaped, and bifid types—emphasizing morphometrics but without integrating clinical or demographic factors.

In contrast, the classification proposed herein introduces a five-type model that synthesizes anatomical, imaging, and clinical data. It accounts for muscle belly number (Types I–III), accessory muscle bands (Type IV), and atypical insertion sites (Type V), providing a more comprehensive and functionally relevant tool for surgical planning, aesthetic evaluation, and anatomical education.

Based on the cumulative anatomical and radiological findings, a comprehensive morphological classification of the zygomaticus muscles is proposed (Table 4). This classification accounts for structural complexity, insertion variability, and clinical relevance, including the estimated prevalence of each variant. Such a framework is intended to guide clinicians in both diagnostic interpretation and procedural planning, particularly in facial aesthetic and reconstructive interventions.

To contextualize the proposed classification system, Table 5 provides a comparative overview of previously published classifications by Pessa, Phan and Onggo, and Elvan, highlighting key structural and clinical distinctions.

## 5. Biomechanics and Function of the Zygomatic Muscles

### 5.1. Smile Kinematics: Muscle Force, Movement Vectors, and Synchronization with Other Facial Muscles (Levator Anguli Oris, Risorius)

The biomechanical function of ZMa and ZMi muscles plays a pivotal role in facial expression, particularly in generating and modulating the human smile. The ZMa is recognized as a key muscular component responsible for elevating and laterally drawing the oral commissure, thereby contributing significantly to smile formation [16,29,59]. The contraction force and vector of the ZMa muscle predominantly influence the characteristic upward and outward movement of the oral commissure, creating a harmonious and aesthetically pleasing smile [9,29,58].

Biomechanical analyses using electromyography (EMG) and three-dimensional (3D) motion capture technologies have demonstrated that the ZMa muscle exerts substantial pulling forces directed superolaterally, with considerable variability influenced by individual anatomical morphology [59,60,61]. Variations in the muscular structure, such as bifid or fan-shaped forms, significantly alter the trajectory of muscular action, thus impacting both the aesthetic and functional aspects of facial expressions [1,55].

Importantly, the ZMa and ZMi muscles do not function in isolation. The biomechanical efficiency of these muscles relies upon synchronized activation with adjacent facial muscles, notably the levator anguli oris (LAO) and risorius muscles [16,61]. The LAO primarily elevates the corner of the mouth vertically, complementing the oblique pull of ZMa, while the risorius muscle contributes horizontal tension, producing a wider, more pronounced smile [25,59,60]. EMG studies confirm coordinated muscular synergy, revealing precise timing and synchronization of ZMa, LAO, and risorius activation patterns during smile production, which are essential for achieving symmetry and balanced facial expressions [25,61].

Understanding these biomechanical relationships is crucial for clinical and surgical practices, including reconstructive procedures and aesthetic interventions aimed at restoring or enhancing facial expressivity. Precise knowledge of muscle force vectors and intermuscular synchronization patterns assists clinicians in achieving optimal functional and aesthetic outcomes in facial reconstructive surgery, cosmetic enhancement procedures, and rehabilitation treatments after facial nerve injuries or paralysis [59,60,61].

### 5.2. Functional Analysis of Smile Types

#### 5.2.1. Duchenne Versus Non-Duchenne Smile

Functional analyses have categorized smiles predominantly into two major types: Duchenne and non-Duchenne smiles. The Duchenne smile is characterized by the simultaneous activation of ZMa muscles and the orbicularis oculi (OOc) muscles, resulting in elevation of the oral commissures along with the presence of characteristic periorbital wrinkles, often termed “crow’s feet” [12,59,62]. Duchenne smiles typically indicate genuine positive emotions and spontaneous affective expression, reflecting authentic emotional engagement [8,12]. EMG studies have consistently confirmed the coordinated activation pattern between ZMa and OOc in producing genuine emotional smiles [8].

In contrast, non-Duchenne smiles involve activation predominantly limited to the ZMa muscles without significant engagement of the OOc, frequently interpreted as polite, social, or posed smiles lacking emotional authenticity [12,59,62]. The functional distinction between these smile types has clinical and psychological implications, notably in social psychology, facial rehabilitation, and aesthetic medicine, as clinicians and researchers frequently differentiate genuine and social smiles based on muscle activation patterns [8,59,62].

#### 5.2.2. Symmetrical Versus Asymmetrical Smile—Influence of Muscle Morphology

The symmetry of the smile is another critical functional dimension significantly influenced by individual anatomical variability in ZMa and ZMi muscles. Symmetrical smiles are generally perceived as aesthetically pleasing and indicative of good facial motor coordination, while asymmetrical smiles may suggest underlying anatomical variability, functional imbalance, or neuromuscular pathologies [25,60,61].

Variations in muscle morphology such as bifid or multiple-bellied ZMa muscles, atypical insertion patterns, or uneven muscle mass between the left and right facial sides can directly contribute to asymmetrical activation during smiling [29,54,58]. For example, bifid ZMa muscles, due to their split muscular bellies, often result in asymmetric muscular tension and differential vectors of movement, potentially creating or enhancing facial dimples and asymmetrical facial expressions [29,58]. Clinical studies have demonstrated that careful consideration of these anatomical differences during reconstructive or aesthetic procedures is crucial in optimizing smile symmetry and overall facial harmony [25,60,61].

In summary, comprehensive functional analysis of different smile types, considering Duchenne and non-Duchenne characteristics as well as symmetrical versus asymmetrical smile patterns, significantly enhances our understanding of facial biomechanics and aesthetics. Such knowledge is fundamental for clinicians and surgeons aiming to improve facial reconstruction, aesthetic outcomes, and the therapeutic management of facial expression disorders [7,8,12,57,58,59,62].

### 5.3. EMG Studies—Muscle Activity Patterns During Facial Expressions

EMG is widely employed to objectively analyze muscle activity patterns during facial expressions, providing critical insights into the functional roles of ZMa and ZMi muscles. EMG enables the quantification and temporal characterization of muscle activation, capturing the precise onset, intensity, duration, and synchronization of muscular contractions during emotional expressions, such as smiling [59,63,64].

Several EMG studies have demonstrated distinctive activity patterns of the ZMa, particularly emphasizing its role in generating genuine emotional smiles (Duchenne smiles). These studies consistently show coordinated activation between ZMa and orbicularis oculi muscles (OOc), characterized by near-simultaneous muscle engagement during spontaneous expressions of positive emotions [12,18,59]. Conversely, non-Duchenne smiles typically show isolated ZMa activation without substantial OOc involvement, allowing clear differentiation between genuine and posed expressions based on EMG recordings [7,8,12,62].

In addition, EMG analyses have highlighted the variable and complex interplay between ZMa, ZMi, and other midface muscles, such as levator labii superioris, levator anguli oris, and risorius, during various emotional displays [61,64]. EMG data also illustrate significant asymmetries and variability in muscle activation patterns, reflecting individual anatomical variations in ZMa and ZMi morphology, such as bifid or multibellied forms, as well as differing insertion points and muscle fiber orientations [29,58].

Clinically, EMG studies offer valuable applications in diagnosing and managing conditions involving facial muscle dysfunction or paralysis, allowing clinicians to assess muscle recovery and guide rehabilitation strategies. The accurate EMG-based mapping of muscle activation patterns aids surgeons in optimizing reconstructive and aesthetic outcomes, particularly in procedures aimed at restoring facial symmetry, dynamic expression, and overall functionality [25,60,61].

In summary, EMG studies provide an essential methodological framework for understanding the detailed functional dynamics of ZMa and ZMi muscles, further emphasizing the clinical importance of precise anatomical knowledge in enhancing facial expressivity and therapeutic effectiveness [59,61,63].

### 5.4. Impact of Morphological Variability on Smile Dynamics and Functional Asymmetry

Morphological variations in the ZMa and ZMi muscles significantly influence smile dynamics, resulting in functional asymmetry and variations in facial expressivity. Diverse anatomical characteristics, such as muscle shape (ribbon-like, fan-shaped, bifid), the presence of accessory muscle bands, atypical insertions, and asymmetry in muscle bellies, directly contribute to differences in the movement trajectory, force production, and symmetry during smiling [1,29,58].

Specifically, the presence of bifid or double-bellied ZMa has been associated with asymmetric muscle activation and directional movement vectors, thereby affecting smile aesthetics and symmetry [29,58]. The inferior belly of the bifid ZMa muscle, often inserting directly into the skin of the cheek, can enhance the visibility of facial dimples during smiling but may also contribute to noticeable functional asymmetry [29,58].

Studies utilizing EMG and three-dimensional facial imaging have confirmed that morphological variations in facial musculature alter functional muscle activation patterns, with variations in insertion points and muscle shape correlating with measurable differences in smile symmetry and dynamic facial expressivity [59,60,61,63]. Individuals with anatomical asymmetry or atypical muscle morphology may demonstrate persistent functional asymmetry, often visible as differences in the amplitude and speed of oral commissure elevation during spontaneous or posed smiles [25,61].

The clinical implications of these morphological influences are significant, especially in aesthetic medicine and reconstructive surgery. The preoperative recognition of anatomical variants such as bifid or accessory muscle bands and atypical insertion sites can guide surgical technique adjustments, minimizing the risk of postoperative asymmetry and optimizing functional outcomes. Accurate knowledge of morphological variability is essential for facial reanimation procedures following nerve injuries or congenital facial asymmetries, enabling precise surgical intervention and rehabilitation strategies [59,60].

In conclusion, the substantial impact of morphological variability in ZMa and ZMi muscles on smile dynamics and functional asymmetry highlights the importance of personalized anatomical assessment for improving clinical, aesthetic, and functional outcomes in facial surgery and rehabilitation [1,25,29,54,58,61].

## 6. Modern Imaging Methods and Functional Assessment

### 6.1. Magnetic Resonance Imaging (MRI)

#### 6.1.1. High-Resolution Imaging for Analysis of Muscle Structure

High-resolution MRI is an advanced non-invasive technique increasingly utilized for the detailed anatomical and structural assessment of facial muscles, including ZMa and ZMi. MRI provides superior soft-tissue contrast, enabling the accurate delineation of individual muscle fibers, anatomical boundaries, and internal morphology, significantly enhancing the understanding of anatomical variations [21,56,65]. Recent studies have employed ultra-high-field MRI scanners (e.g., 3T or 7T) to visualize subtle muscular differences, such as bifid and accessory muscle bellies, atypical insertions, and intricate relationships with adjacent structures [1,28,29,58].

Clinically, high-resolution MRI has significant implications for preoperative planning, particularly in aesthetic and reconstructive surgery. Detailed knowledge of the ZMa and ZMi muscle structure allows surgeons to precisely navigate anatomical complexities, reduce complications, and optimize surgical outcomes [21,55,56].

#### 6.1.2. Topographical Mapping of Mimic Muscles at Rest and Activation

MRI-based topographical mapping is a valuable approach for characterizing the spatial distribution and functional behavior of mimic muscles, such as ZMa and ZMi, under resting and active conditions. Functional MRI studies involving dynamic or sequential imaging provide critical insights into muscle movement patterns, contraction intensity, and interaction with neighboring muscles during facial expressions such as smiling [21,61,65]. Such mapping enables clear differentiation between resting muscle morphology and dynamic muscular behavior during various expressions, facilitating a deeper understanding of facial biomechanics [59,61,65].

Dynamic MRI sequences have revealed that variations in ZMa and ZMi structure correlate with distinct functional outcomes, including asymmetric muscle activation and altered expression dynamics [59,61]. Comprehensive MRI-based mapping is particularly beneficial for quantifying the degree of functional asymmetry, essential for diagnosing neuromuscular conditions, planning facial reanimation procedures, and evaluating postoperative outcomes [21,60,61].

Ultimately, the integration of high-resolution MRI and topographical muscle mapping contributes significantly to personalized facial surgery, rehabilitation strategies, and improved aesthetic and functional results in clinical practice [21,56,61,65].

### 6.2. Ultrasonography (US)

#### 6.2.1. Dynamic Ultrasonography as a Tool for Real-Time Functional Analysis

Dynamic US has increasingly become an important imaging modality for real-time functional analysis of facial muscles, including ZMa and ZMi, due to its accessibility, portability, and capability to visualize muscle movements during active facial expressions. The US enables clinicians to observe muscular contraction dynamics and displacement patterns in real time, thereby providing immediate feedback on functional muscle activation [56,66,67].

The primary advantage of dynamic US is its ability to capture live images of muscle activity during various expressions, such as smiling or speaking, thus allowing clinicians to assess muscle contraction speed, amplitude, and synchrony. This functional insight is particularly valuable for identifying subtle asymmetries or impaired muscle coordination that may not be apparent during static imaging techniques [66,67].

Clinically, dynamic US has significant applications in facial rehabilitation, providing a non-invasive method of monitoring therapeutic progress and evaluating recovery after facial nerve injuries, reconstructive surgeries, or cosmetic interventions [56,66,67]. Thus, dynamic US constitutes a highly effective tool for optimizing treatment outcomes and enhancing individualized therapeutic strategies.

#### 6.2.2. Detection of Asymmetry and Additional Muscle Bands

The US is also valuable for detecting morphological asymmetries and identifying accessory muscle bands or variants within ZMa and ZMi muscles. Due to its high-resolution imaging of superficial tissues, US effectively delineates muscle architecture, enabling clinicians to detect anatomical anomalies such as bifid muscles, accessory slips, or duplications that significantly influence facial symmetry and expressivity [1,67].

Studies employing US have demonstrated its utility in identifying the presence and location of additional muscular bands and atypical muscle insertions, as well as their functional consequences in facial asymmetry [1,67]. Detection of these anatomical variations is crucial for individualized preoperative planning, allowing clinicians to anticipate potential functional asymmetries and tailor surgical or aesthetic procedures accordingly [1,56,66].

Overall, dynamic US is a versatile imaging modality for the real-time functional assessment and detailed anatomical characterization of ZMa and ZMi, significantly enhancing clinical diagnostics, surgical precision, and therapeutic management in facial medicine and reconstructive surgery [1,56,66,67].

### 6.3. Three-Dimensional Facial Scanning and Photogrammetry

#### 6.3.1. Evaluation of Facial Expressions in Motion and Computer Modeling

Three-dimensional (3D) facial scanning and photogrammetry have emerged as powerful tools for the dynamic analysis and computer modeling of facial expressions, providing detailed visualization and quantification of facial movements associated with muscle activation, including ZMa and ZMi muscles [3,65,68,69]. These methods allow the precise capturing and reconstruction of facial surfaces in real time, enabling the assessment of subtle changes and movements during expressions such as smiling or speaking [65,70].

Computer-generated models derived from 3D facial scans effectively visualize and quantify muscle-generated facial deformation, thus providing detailed biomechanical insights into individual muscular function and interplay [68,70]. This capability is particularly useful in characterizing the functional impact of anatomical variations, including muscle asymmetry, bifid structures, and accessory muscle bands, on facial expression and aesthetics [68,69,70].

#### 6.3.2. Potential Application in Surgical Planning and Facial Prosthetics

The use of 3D facial scanning and photogrammetry holds significant potential in surgical planning and facial prosthetics by providing accurate, individualized anatomical data critical for reconstructive and aesthetic interventions. High-fidelity 3D models assist surgeons in preoperatively evaluating the spatial relationships of ZMa and ZMi muscles with adjacent structures, optimizing surgical approaches, and predicting postoperative outcomes [65,68,70].

In facial prosthetics and reconstructive surgery, computer-generated 3D facial models facilitate the precise fabrication of customized prosthetic devices, improving anatomical fit, functional integration, and overall aesthetic outcomes. Moreover, 3D photogrammetry offers valuable insights into postoperative facial symmetry and function, enhancing patient-specific rehabilitation strategies and therapeutic interventions [68,69,70].

In conclusion, the incorporation of 3D facial scanning and photogrammetry into clinical practice represents a significant advancement in personalized facial surgery and prosthetics, enabling detailed functional assessment and improving surgical precision and patient outcomes [65,68,69,70].

### 6.4. Artificial Intelligence (AI)

#### 6.4.1. Muscle Segmentation in Imaging Studies

Artificial intelligence (AI) techniques, particularly machine learning (ML) and deep learning (DL), have significantly advanced the segmentation and detailed analysis of facial muscles, including ZMa and ZMi, in imaging studies. Recent AI applications enable the automatic, rapid, and precise delineation of muscle structures from MRI, US, and 3D imaging datasets, substantially improving anatomical visualization and diagnostic accuracy [3,66,71].

AI-driven segmentation algorithms effectively distinguish subtle anatomical boundaries between facial muscles, accurately identifying complex anatomical variants, accessory muscle bands, bifid muscles, and asymmetric features previously challenging to detect reliably through conventional methods [66,71]. Such advancements enhance diagnostic precision, facilitate consistent inter-individual comparisons, and optimize preoperative planning for reconstructive and aesthetic procedures [3,71].

#### 6.4.2. Automatic Classification of Morphological Variants—An Emerging Tool

AI-based automated classification systems represent an emerging tool for systematically categorizing morphological variants of ZMa and ZMi muscles. Machine learning algorithms trained on extensive imaging datasets have demonstrated the capability to recognize and classify distinct anatomical patterns, including bifid or multiple-bellied muscle forms, atypical insertions, and other clinically relevant morphological variations [3,66,71].

Automated classification through AI provides clinicians with rapid, reproducible, and accurate identification of anatomical variations, directly contributing to personalized medical and surgical management strategies. Such tools significantly reduce observer variability, enhance diagnostic efficiency, and support clinical decision-making, particularly in complex surgical scenarios or facial rehabilitation planning [3,66].

A wide range of imaging modalities has been applied to the evaluation of the ZMa and ZMi, each offering specific advantages in terms of resolution, dynamic assessment, and clinical applicability. Table 6 summarizes the key characteristics, benefits, and limitations of each imaging approach discussed in this review.

Recent advances in artificial intelligence (AI) have facilitated more precise segmentation and morphometric analysis of facial muscles, including the ZMa and ZMi. Among the most commonly utilized platforms is 3D Slicer, an open-source software that supports multimodal data integration, including MRI and CT, and allows for the semi-automated segmentation of soft tissues [13]. Additionally, DeepLabCut has emerged as a powerful tool for markerless pose estimation and muscle tracking, with recent extensions into human musculoskeletal modeling [72].

Despite these advancements, AI-based methods are not without limitations. Rare anatomical variants, like multibellied ZMa or atypical insertional patterns, are often underrepresented in training datasets, which can lead to misclassification or segmentation errors. Furthermore, deep learning models require extensive annotation by domain experts, and their diagnostic utility may vary depending on population-based variability or imaging quality [73].

Improved training sets incorporating cadaveric, radiological, and photogrammetric data, as proposed in our forthcoming original study, may help overcome these challenges by increasing anatomical diversity and fidelity in machine learning applications.

## 7. Anthropological Significance and Reconstructive Applications

### 7.1. Role of ZMa and ZMi in Forensic Facial Reconstruction Based on Skeletal Remains

ZMa and ZMi muscles play a critical role in forensic facial reconstruction, where soft-tissue features are reconstructed from skeletal remains. The accurate anatomical placement, morphology, and volumetric estimation of these muscles are fundamental to reliably recreate facial appearance, expression, and individuality from skull morphology [20,74,75]. Morphological variations, such as bifid muscle structures, additional muscle bands, or atypical insertions, significantly influence the final appearance of reconstructed faces, especially affecting expression nuances such as smiles or subtle facial movements [20,75]. In addition to individual anatomical differences, population-level variables such as sex, age, and ethnicity have been shown to influence the morphology of the zygomaticus muscles. These differences are essential in forensic and surgical contexts, as they affect the accuracy of facial reconstructions and aesthetic outcomes. Table 7 presents a summary of the most relevant demographic factors, their anatomical effects, and clinical relevance.

### 7.2. Morphological Variability as a Parameter in Population Analyses and Forensic Anthropology

Morphological variability in the ZMa and ZMi muscles provides essential anthropological data for population-based analyses and forensic anthropology. Distinctive muscular variants, such as the presence of bifid ZMa, have shown population-specific frequencies, making them valuable markers for estimating ancestry or population affinity [54,58]. These anatomical variations, detectable through forensic facial reconstruction, can aid forensic anthropologists in making more precise estimations regarding individual identity, ethnicity, and population affiliation in forensic contexts [20,54,74,75].

### 7.3. Influence of Muscle Morphology on Aesthetics and Expression in Historical Reconstructions (Museums, Art)

The morphology and anatomical positioning of ZMa and ZMi muscles have significant implications in historical facial reconstructions used in museums, archaeological contexts, and artistic representations. Accurate portrayal of these muscles is vital in reproducing authentic facial expressions, such as smiles or subtle emotional states, thus providing viewers with realistic insights into the appearance and emotional expressions of historical individuals [20,75]. Understanding morphological variability and its influence on facial aesthetics ensures historically accurate reconstructions, enhancing both educational value and realism in museum displays and artistic renderings [20].

In conclusion, the anthropological significance of ZMa and ZMi muscles extends across forensic reconstruction, population studies, and historical recreations, underlining the importance of comprehensive anatomical understanding in reconstructive and anthropological contexts [20,54,58,74,75].

In conclusion, AI-driven muscle segmentation and the automatic classification of morphological variants are promising tools that enhance anatomical and clinical understandings of ZMa and ZMi muscles, significantly improving diagnostic accuracy, procedural planning, and patient-specific treatment outcomes [3,66,71].

While the zygomaticus muscles serve as important landmarks for estimating facial soft-tissue thickness (FSTT), it is critical to recognize the inherent limitations in facial reconstruction based solely on skeletal remains. Without soft-tissue data, reconstructions rely heavily on population-based averages, which may not capture individual variability. Recent research [14] highlights that incorporating zygomatic landmarks improves regression models for FSTT estimation adjusted for age, sex, and BMI; however, significant variability remains, underscoring the tentative nature of these reconstructions.

## 8. Discussion

The ZMa and ZMi have long been described as key contributors to facial expression, particularly smiling. However, the extensive morphological variability observed in recent anatomical and imaging studies challenges the traditional simplistic binary understanding of their structure and calls for a more nuanced classification and clinical interpretation.

### 8.1. Clinical Relevance of the Proposed Classification

The proposed five-type morphological classification (see Table 3) provides a clinically oriented framework for understanding the diversity of ZMa/ZMi. While Type I (standard) represents the typical anatomy described in classical texts [16], the prevalence of Type II (bifid) ZMa—ranging from 13% to 34% in the general population [29,54]—suggests that this variant is not a mere anomaly but a significant and recurring phenotype. Its association with facial dimples and asymmetric smiling has implications for both aesthetic procedures and the diagnosis of neuromuscular disorders.

Type III (multibellied) and Type IV (accessory bands) add further complexity to clinical interpretation. These variations may obscure standard anatomical landmarks, complicate surgical interventions (e.g., midface lifts or smile reanimation procedures), and affect the predictability of botulinum toxin injection outcomes [1]. In contrast, Type V (atypical insertions) may directly alter facial expressivity and are often encountered in reconstructive or forensic settings [20,55].

### 8.2. Morphological Variability and Facial Biomechanics

From a functional perspective, morphological variants likely influence force vectors, insertion angles, and dynamic movement patterns during facial expressions. Muscles with multiple bellies or atypical insertions could generate divergent trajectories, leading to subtle differences in emotional signaling or perceived facial symmetry [21,61]. Despite this, the relationship between morphology and biomechanics remains insufficiently explored, particularly in vivo.

### 8.3. Imaging and Identification Challenges

Although MRI and US allow the increasingly precise visualization of facial musculature, they are limited in detecting fine slips or accessory fibers, especially in dynamic contexts [66,67]. Innovations in AI-based imaging and 3D photogrammetry have shown promise in delineating variant morphologies and predicting symmetry in reconstructive planning [68,71]; however, these tools still rely heavily on manually annotated databases and lack widespread clinical integration.

### 8.4. Developmental and Evolutionary Considerations

The developmental basis of ZMa/ZMi variability may be linked to neural crest cell migration and craniofacial mesodermal patterning [27]. Genetic influences, such as those associated with syndromes like Williams or Treacher Collins, offer further insights into aberrant muscle development and facial morphology [45,46]. From an evolutionary perspective, inter-individual differences in smiling musculature may reflect adaptations in emotional communication, particularly in complex social species like Homo sapiens [4].

### 8.5. Limitations and Future Directions

This study is primarily anatomical and descriptive. Further research should incorporate EMG, biomechanical simulation, and genetic analysis to establish causal links between muscle morphology and facial function. Moreover, a population-wide meta-analysis could reveal ethnogeographic trends in variant prevalence and functional consequences.

## 9. Conclusions

A revised anatomical framework for ZMa and ZMi, as proposed here, enhances both scientific understanding and clinical precision. Integrating anatomical, functional, and population-based insights may improve surgical outcomes, personalize aesthetic procedures, and deepen our understanding of facial expression and identity.

## Figures and Tables

**Table 1:** Classical anatomy of zygomatic muscles.

Muscle	Origin	Insertion	Innervation	Blood Supply	Main Function
ZMa	Zygomatic bone (lateral surface)	Modiolus (corner of the mouth)	Facial nerve (CN VII)	Facial artery	Pulling corners of mouth upward and outward (smile)
ZMi	Zygomatic bone (anterior aspect)	Upper lip, skin near nasolabial fold	Facial nerve (CN VII)	Facial artery	Elevating upper lip (smile, subtle expressions)

**Table 2:** Summary of genetic and syndromic conditions affecting zygomaticus muscle morphology.

Gene/Syndrome	Associated Effect on ZMa/ZMi Morphology
Tbx1	Disruption linked to craniofacial hypoplasia, abnormal muscle formation
MyoD, Myf5	Critical in early myogenesis; mutations impair muscle segmentation and positioning
Möbius syndrome	Facial paralysis; ZMa/ZMi underdeveloped or absent
Myotonic dystrophy type 1	Progressive muscle atrophy; ZMa visibly reduced on MRI
Facioscapulohumeral dystrophy	Weakness and asymmetry of facial muscles, including ZMa

**Table 3:** Main morphological variants of ZMa and ZMi muscles.

Variant Type	Description	Clinical Relevance	Prevalence	Literature Sources
Bifid ZMa	Muscle splitting into two bellies (superior/inferior)	Associated with facial dimples, asymmetry in smiling	~13–34%	Pessa et al., 1998 [29]; Phan and Onggo, 2019 [54]
Multibellied ZMa	Presence of more than two bellies	Influences expression symmetry	Rare	Elvan et al., 2020 [1]
Accessory ZMi bands	Additional bands duplicating typical ZMi	Potential impact on surgical outcomes, injection techniques	Occasionally observed	Spiegel and DeRosa, 2005 [55]
Atypical insertions	Insertions into upper lip, orbicularis oris, or cheek skin	Affects facial expressivity and surgical procedures	Common	Elvan et al., 2020 [1]; Spiegel and DeRosa, 2005 [55]

**Table 4:** Proposed classification of zygomatic muscle variants (updated with prevalence).

Category	Description	Prevalence	Clinical Importance
Type I—single belly	Typical anatomy of ZMa/ZMi		Standard reference for surgery
Type II—double belly	Clearly separated superior/inferior bellies	~13–34%	Associated with dimples, aesthetic implications
Type III—multibellied	More than two distinct bellies	Rare	Increased complexity in surgical management
Type IV—accessory bands	Presence of additional muscle slips	Occasionally observed	Potential effect on mimic expression and symmetry
Type V—atypical insertion	Insertions into non-typical anatomical points	Common	Impacts surgical outcomes and expression dynamics

**Table 5:** Comparison of zygomaticus muscle classifications.

Author	Year	Classification Basis	Limitations/Scope
Pessa et al. [29]	1998	Binary: presence or absence of bifid ZMa	Limited to bifid variant only
Phan and Onggo [54]	2019	Meta-analysis of bifid ZMa prevalence	Focused on population prevalence, limited to bifid
Elvan et al. [1]	2020	Shape-based: ribbon-like, fan-shaped, bifid	Lacks clinical correlation and demographic factors
Present Study	2025	Five-type classification: muscle belly count, accessory bands, insertion types, population variability	Comprehensive; includes anatomy, imaging, and clinical relevance

**Table 6:** Imaging methods for ZMa and ZMi muscles.

Imaging Modality	Advantages	Limitations	Clinical Application	Literature Sources
MRI (high-resolution)	Excellent soft-tissue contrast, non-invasive, 3D analysis	Costly, limited accessibility, static or limited dynamic analysis	Preoperative anatomical mapping	Cotofana et al., 2018 [21]
Dynamic Ultrasonography	Real-time muscle visualization, affordable, non-invasive	Limited depth resolution	Functional analysis, dynamic asymmetry detection	Kwon et al., 2014; Lee et al., 2020 [33]
3D Photogrammetry	Precise facial surface modeling, dynamic analysis	Superficial only, requires specialized equipment	Surgical planning, prosthetic fitting	Knoops et al., 2017 [68]
AI-based Imaging Analysis	Fast, objective, accurate identification of variants	Requires training dataset, computational resources	Automated diagnostics, clinical classification of variants	Lee et al., 2021 [33]; Codari et al., 2020

**Table 7:** Population variability in ZMa and ZMi muscles.

Variable	Key Findings	Clinical Implications	Literature Sources
Sex	Males: typically larger, thicker muscles; females: thinner, smaller volume	Aesthetic procedures, individualized planning	Standring, 2021; Loukas et al., 2006 [21]
Age	Progressive muscle atrophy, reduced elasticity	Impact on facial expressions, surgical rejuvenation outcomes	Spiegel and DeRosa, 2005 [55]; Cotofana et al., 2018 [21]
Ethnicity	Variability in prevalence of bifid muscles (higher in Asian and American populations)	Consideration in forensic identification, surgical approaches	Phan and Onggo, 2019 [54]; Hu et al., 2008 [58]

## List of Abbreviations

Abbreviation	Full Term
ZMa	Zygomaticus major
ZMi	Zygomaticus minor
SMAS	Superficial musculoaponeurotic system
EMG	Electromyography
MRI	Magnetic resonance imaging
US	Ultrasonography
CN VII	Cranial nerve VII (Facial nerve)
FGF	Fibroblast growth factor
Shh	Sonic hedgehog
AI	Artificial intelligence
3D	Three-dimensional
Tbx1, MyoD, Pax3, Pitx2	Key transcription factors in facial muscle development
